# Occurrence and Reduction of Biogenic Amines in Kimchi and Korean Fermented Seafood Products

**DOI:** 10.3390/foods8110547

**Published:** 2019-11-04

**Authors:** Young Kyoung Park, Jae Hoan Lee, Jae-Hyung Mah

**Affiliations:** Department of Food and Biotechnology, Korea University, 2511 Sejong-ro, Sejong 30019, Korea; eskimo@korea.ac.kr (Y.K.P.); jae-lee@korea.ac.kr (J.H.L.)

**Keywords:** kimchi, *Jeotgal*, *Aekjeot*, *Myeolchi-jeot*, *Myeolchi-aekjeot*, biogenic amines, recommended limits, occurrence, reduction, starter cultures

## Abstract

Biogenic amines produced during fermentation may be harmful when ingested in high concentrations. As current regulations remain insufficient to ensure the safety of fermented vegetable products, the current study determined the risks associated with the consumption of kimchi by evaluating the biogenic amine concentrations reported by various studies. Upon evaluation, some kimchi products were found to contain histamine and tyramine at potentially hazardous concentrations exceeding the recommended limit of 100 mg/kg for both histamine and tyramine. The biogenic amines may have originated primarily from metabolic activity by microorganisms during fermentation, as well as from *Jeotgal* (Korean fermented seafood) and *Aekjeot* (Korean fermented fish sauce) products commonly used as ingredients for kimchi production. Many studies have suggested that *Jeotgal* and *Aekjeot* may contribute to the histamine and tyramine content in kimchi. Microorganisms isolated from kimchi and *Jeotgal* have been reported to produce both histamine and tyramine. Despite the potential toxicological risks, limited research has been conducted on reducing the biogenic amine content of kimchi and *Jeotgal* products. The regulation and active monitoring of biogenic amine content during kimchi production appear to be necessary to ensure the safety of the fermented vegetable products.

## 1. Introduction

**Figure 1:**
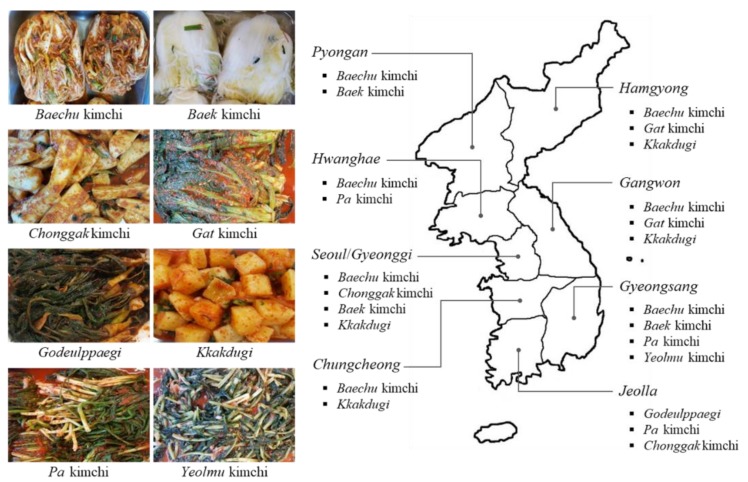
Kimchi varieties available across different provinces in Korea. *Baechu* kimchi: Napa cabbage kimchi; *Baek* kimchi: Napa cabbage kimchi prepared without red pepper powder; *Chonggak* kimchi: ponytail radish kimchi; *Gat* kimchi: mustard leaf kimchi; *Godeulppaegi*: Korean lettuce kimchi; *Kkakdugi*: diced radish kimchi; *Pa* kimchi: green onion kimchi; *Yeolmu* kimchi: young radish kimchi.

Kimchi refers to a group of traditional Korean fermented vegetable products consumed worldwide [1]. Dating back to the 12th century during the Three Kingdoms period of ancient Korea, salted and fermented vegetable products represent the earliest form of kimchi, however, the addition of several ingredients such as the introduction of red peppers in the 16th century was eventually adopted for kimchi production [2]. The availability of local ingredients across different provinces in Korea led to the development of many regional kimchi varieties [3] (Figure 1). Currently, there are over 200 varieties of kimchi with over 100 different ingredients used for kimchi production [4]. Each kimchi variety is categorized according to the ingredients selected for production [5]. Kimchi in its current form has been recognized globally through international standardization as well [6]. Kimchi is prepared by trimming Napa cabbage, followed by salting, rinsing, and then draining excess water. The seasoning ingredients include red pepper powder, garlic, ginger, radish, glutinous rice paste, sugar, *Jeotgal*, and *Aekjeot*. The salted Napa cabbage is then mixed with the seasoning and stored at low temperatures (typically 0–10 °C in Korea [5]) to ferment until ripened [6]. While the production method described by the Codex only describes *Baechu* kimchi (Napa cabbage kimchi), slight variations are used to produce other kimchi varieties.

Nonetheless, nearly every kimchi variety benefits from preliminary brining, which inhibits the growth of pathogenic bacteria while selecting for lactic acid bacteria (LAB) known for promoting beneficial effects such as gastrointestinal regulation and prevention of colon cancer [7,8]. The LAB such as *Leuconostoc*, *Lactobacillus*, and *Weissella* species as well as the enzymes present in the ingredients are responsible for kimchi fermentation [9,10]. Consumption of kimchi is reported to provide numerous health benefits such as anti-oxidative, anti-carcinogenic, anti-mutagenic, and anti-aging effects [8,11,12].

Despite the numerous beneficial functional qualities, fermented foods such as kimchi may contain potentially harmful substances known as biogenic amines (BA). The nitrogenous compounds are mostly produced by microorganisms during fermentation through enzymatic decarboxylation of amino acids, as well as transamination of ketones and aldehydes [13]. BA are often categorized as aliphatic: putrescine, cadaverine, spermidine, spermine; aromatic: *β*-phenylethylamine, tyramine; heterocyclic: tryptamine, histamine [14,15]. The intake of BA at high concentrations as well as amine oxidase inhibition and deficiency may lead to toxic effects [16]. Recently, histamine, tyramine, putrescine, cadaverine, spermidine, and spermine were found to be cytotoxic toward human intestinal cells [17,18,19]. Furthermore, BA may also be converted to potentially carcinogenic N-nitrosamines in the presence of nitrites [20,21]. Excessive intake of foods containing high concentrations of histamine may potentially induce “scombroid poisoning” with symptoms such as headaches, hives, diarrhea, dyspnea, and hypotension [22]. Similarly, ingestion of foods with excessive tyramine content may cause a “cheese crisis” with symptoms that include severe headaches, hemorrhages, hypertensive effects or even heart failure [23]. As a result, many countries have implemented regulations on the production of histamine-rich seafood products, however many other food products are not currently regulated [24]. Several studies have suggested limits for BA content in food products of 100 mg/kg for histamine, 100–800 mg/kg for tyramine, 30 mg/kg for *β*-phenylethylamine, and 1000 mg/kg for total BA content [14,15]. The concentrations of BA in many fermented food products such as fermented meats and cheese have been widely reported to exceed limits for safe consumption. Similarly, BA have been detected in kimchi products, the most widely consumed traditional Korean food. High concentrations of BA have also been detected in kimchi ingredients *Jeotgal* (Korean fermented seafood) and *Aekjeot* (Korean fermented fish sauce), which contribute to the overall BA content in kimchi [25]. In addition, microorganisms isolated from kimchi as well as the fermented seafood products *Jeotgal* and *Aekjeot* have been reported to produce BA. Current regulations remain insufficient to address the potential health risks associated with the consumption of kimchi with high concentrations of BA. Therefore, the current article evaluated the risks associated with the BA content of kimchi products according to intake limits for *β*-phenylethylamine (30 mg/kg), histamine (100 mg/kg), and tyramine (100 mg/kg) as recommended by Ten Brink et al. [15], and reviewed potential sources of BA, and methods for reducing BA content.

## 2. Biogenic Amine Content in Kimchi Products

Table 1 displays the BA content of kimchi products as reported by various studies. The BA content of *Baechu* kimchi (Napa cabbage kimchi), the most popular kimchi variety consumed worldwide, has been reported by several studies. Cho et al. [25] reported histamine and tyramine concentrations in *Baechu* kimchi that exceeded recommended limits. Another study also showed that tyramine content in *Baechu* kimchi exceeded the recommended limit [26]. Tsai et al. [27] notably reported the highest histamine content which exceeded the recommended limit by a factor of 53. Tsai et al. [27] suggested that the high concentration of histamine in kimchi might be due to ingredients such as fish sauce or shrimp paste used in the kimchi production process. Shin et al. [28] reported *β*-phenylethylamine, histamine, and tyramine content at safe concentrations below 30 mg/kg. Similarly, Mah et al. [29] reported both histamine and tyramine content at safe concentrations below 30 mg/kg. In ripened *Baechu* kimchi, Kang et al. [26] reported tyramine content at concentrations that nearly reached the recommended limit.

Aside from *Baechu* kimchi, several studies have also reported the BA content of other kimchi varieties as well. *Chonggak* kimchi (ponytail radish kimchi) as reported by Jin et al. [30] contained histamine concentrations that exceeded the recommended limit. Tyramine content in *Chonggak* kimchi as reported by Kang et al. [26] were at safe concentrations, while Mah et al. [29] reported safe concentrations of both histamine and tyramine below recommended limits. As for *Gat* kimchi (mustard leaf kimchi), Lee et al. [31] reported histamine concentrations which exceeded the recommended limit by a factor of 2, while tyramine content slightly exceeded the limit. In contrast, Mah et al. [29] reported that *Gat* kimchi did not contain histamine and tyramine at detectable levels. *Kkakdugi* (diced radish kimchi) as reported by Jin et al. [30] contained tyramine at safe concentrations below the recommended limit, however, histamine concentrations exceeded the recommended limit. In contrast, Mah et al. [29] reported that histamine was not detected in *Kkakdugi*, and tyramine content was at safe concentrations below recommended limits. Similarly, Kang et al. [26] also reported tyramine concentrations in *Kkakdugi* below the recommended limit. As for *Pa* kimchi (green onion kimchi), Lee et al. [31] reported histamine and tyramine concentrations exceeded recommended limits by a factor of 4 and 2, respectively. In contrast, Mah et al. [29] reported histamine and tyramine in *Pa* kimchi at safe concentrations as tyramine content was not detected while histamine content remained below 30 mg/kg. Other kimchi varieties such as *Baek* kimchi (Napa cabbage kimchi prepared without red pepper powder), *Godeulppaegi* (Korean lettuce kimchi), and *Yeolmu* kimchi (young radish kimchi) were reported to contain histamine and tyramine at safe concentrations below 100 mg/kg [29].

Nonetheless, as the vast majority of studies are primarily focused upon *Baechu* kimchi, further research on the BA content of other kimchi varieties remains necessary. Currently, the severity of the risks associated with the BA content of kimchi remains difficult to thoroughly assess as limited research has been conducted. Though various BA have been detected in kimchi products, several studies have reported histamine and tyramine content at concentrations that exceeded the recommended intake limits of 100 mg/kg. Furthermore, the risk of nitrosamine formation entails the need for continuous monitoring of BA content during fermentation, especially as putrescine and cadaverine were detected at particularly high concentrations. Due to the toxicological risks associated with the consumption of BA, the content in kimchi necessitates regulation and control to ensure its safety.

## 3. Biogenic Amine Content of Other Vegetable Products

Research has also been conducted on the BA content of vegetable products originating from other countries (Table S1). The popular fermented food sauerkraut is produced through lactic acid fermentation of white cabbage [32,33]. Among European fermented food products, sauerkraut most closely resembles Korean kimchi [34]. Despite its popularity, Taylor et al. [35] reported that sauerkraut contained histamine concentrations that exceeded recommended limits. Ten Brink et al. [15] also reported that histamine and tyramine in sauerkraut exceeded recommended limits by a factor of 1 and 2, respectively. Many varieties of Japanese *Tsukemono* are preserved vegetables produced utilizing methods such as fermentation, salting, and pickling [36]. *Tsukemono* are differentiated based on ingredients, pickling method, and microorganisms responsible for fermentation [5]. Handa et al. [37] reported that histamine and tyramine in *Tsukemono* exceeded recommended limits by a factor of 3 and 4, respectively. As an important part of the Taiwanese diet, mustard pickle is prepared using mustard greens submerged in 14% NaCl brine for 4 months [38]. Kung et al. [38] reported that mustard pickles contained histamine and tyramine at safe concentrations below 100 mg/kg. Though fermented vegetable products are consumed worldwide, limited research has been conducted on the BA content of vegetable-based fermented foods. The few studies available had reported a wide range of BA content, including concentrations that exceeded recommended limits. Therefore, as the risks associated with the consumption of fermented vegetables remains largely undetermined, additional research is necessary to ensure the safe consumption of fermented foods.

## 4. Determinants for Biogenic Amine Content in Kimchi

### 4.1. Biogenic Amine Content of Kimchi Ingredients: Jeotgal and Aekjeot

Kimchi production involves the use of many ingredients including the fermented seafood products *Jeotgal* and *Aekjeot*. Used as seasoning ingredients during the production of kimchi [39], *Jeotgal* and *Aekjeot* contain flavor compounds that contribute greatly to the ripening process during kimchi fermentation [40]. Reports of the fermented seafood products as kimchi ingredients date back to the 16th century during the age of the *Chosun* dynasty of Korea [41]. Though *Jeotgal* and *Aekjeot* used during modern kimchi production vary by region, the most commonly used varieties include *Myeolchi*-*jeot* (salted and fermented anchovy), *Myeolchi*-*aekjeot* (salted and fermented anchovy sauce), *Saeu*-*jeot* (salted and fermented shrimp), and *Kkanari*-*aekjeot* (salted and fermented sand lance sauce) [42]. *Jeotgal* production typically involves submersion of seafood in brine with 20% salinity for 2–3 months at room temperature, and results in the final product resembling the initial seafood ingredient [43]. Some *Jeotgal* products undergo additional seasoning for consumption as side dishes rather than as ingredients during kimchi production [44,45]. Similarly, *Aekjeot* production involves the submersion of seafood in brine with salinity ranging from 20 to 30% for 1–2 years, however solid particles are removed through filtration for the final product [46]. In both *Jeotgal* and *Aekjeot*, the salt content inhibits putrefactive bacteria, and the enzymatic activity partially breaks down the proteins to develop a rich flavor [41]. Also, the addition of *Jeotgal* contributes to the protein, amino acid, and mineral content of kimchi, further reinforcing the nutritional value of kimchi products [5].

Despite the benefits described above, *Jeotgal* and *Aekjeot* have been reported to contain high concentrations of potentially hazardous BA such as histamine and tyramine [29]. Table 2 displays the BA content of the fermented seafood products. The reported BA content of *Aekjeot* and *Jeotgal* were evaluated according to recommended limits for intake. *Myeolchi*-*jeot* was reported to contain histamine and tyramine concentrations which exceeded recommended limits by a factor of approximately 6 and 2, respectively [47]. *Myeolchi*-*aekjeot* reportedly contained histamine and tyramine at concentrations that exceeded recommended limits by a factor of approximately 12 and 4, respectively [29]. The BA content of *Myeolchi*-*aekjeot* as studied by Cho et al. [25] showed *β*-phenylethylamine, histamine, and tyramine content at concentrations that exceeded recommended limits by a factor of about 2, 11, and 6, respectively. Moon et al. [48] also studied the BA content of *Myeolchi*-*aekjeot* by reporting *β*-phenylethylamine, histamine, and tyramine content at concentrations that exceeded recommended limits by a factor of approximately 3, 12, and 4, respectively. Similarly, Shin et al. [28] reported that *Myeolchi*-*aekjeot* contained *β*-phenylethylamine, histamine, and tyramine at concentrations that exceeded recommended limits by a factor of approximately 1, 4, and 4, respectively. Cho et al. [49] and Joung and Min [50] reported histamine concentrations in *Myeolchi*-*aekjeot* which greatly exceeded recommended limits by a factor of about 21 and 11, respectively.

As for *Kkanari*-*aekjeot*, histamine and tyramine content were reported at concentrations that exceeded recommended limits by a factor of approximately 10 and 2, respectively [29]. Cho et al. [25] also reported the *β*-phenylethylamine, histamine, and tyramine content in *Kkanari*-*aekjeot* at concentrations which exceeded recommended limits by a factor of 2, 11, and 6, respectively. Moon et al. [48] reported histamine and tyramine content at concentrations that exceeded recommended limits by a factor of about 7 and 3, respectively. Similarly, Shin et al. [28] reported that *Kkanari*-*aekjeot* contained *β*-phenylethylamine, histamine, and tyramine at concentrations that exceeded recommended limits by a factor of approximately 1, 10, and 3, respectively. Notably, the highest histamine content in *Kkanari*-*aekjeot* was reported by Cho et al. [49] as concentrations greatly exceeded the recommended limit by a factor of approximately 18.

As for *Saeu*-*jeot*, Mah et al. [47], Cho et al. [25], Moon et al. [48], and Shin et al. [28] reported BA content at safe concentrations below recommended limits for *β*-phenylethylamine, histamine, and tyramine, respectively.

Overall, the considerably high BA concentrations, especially histamine, reported for both retail *Jeotgal* and *Aekjeot* products may be potentially hazardous. All *Kkanari*-*aekjeot* and *Myeolchi*-*aekjeot* products contained histamine concentrations which exceeded 100 mg/kg indicating that safety regulations are necessary. According to Mah et al. [29], the high BA content may be due to the considerably long fermentation duration for the production of the fermented seafood. Furthermore, the results of the research conducted by Moon et al. [48] suggested that total BA content increased alongside crude protein concentrations for both *Jeotgal* and *Aekjeot*. After all, the high concentrations of BA reported for kimchi appears to originate partly from fish sauce such as *Myeolchi*-*aekjeot* and *Kkanari*-*aekjeot* [25]. Given the high concentrations of BA detected in kimchi and fermented seafood products, safety regulation and standardization of the manufacturing process appears to be necessary.

High BA concentrations were not limited to *Jeotgal* and *Aekjeot* products as the similar observations were reported for fermented seafood products originating from other countries (Table S1). Saaid et al. [51] studied the BA content of Malaysian seafood. The study showed that *Cincalok* (salted and fermented shrimp) contained histamine and tyramine at high concentrations that exceeded the recommended limits by a factor of approximately 3 and 7, respectively. *Budu* (salted and fermented anchovy) also contained high histamine and tyramine concentrations that exceeded recommended limits by a factor of 4 and 9, respectively. Similarly, research conducted by Rosma et al. [52] revealed histamine concentrations in *Budu* exceeding the recommended limit by a factor of 11.

The reported results indicated that fermented seafood products tended to contain high concentrations of BA, especially histamine. As the BA content exceeded well beyond recommended limits, consumption of the fermented seafood products may lead to adverse effects on human health. Due to the potential toxicological risks, expansion of current regulations regarding the BA content of seafood appears to be necessary to cover the aforementioned fermented seafood products as well as to include other amines such as tyramine and *β*-phenylethylamine.

### 4.2. Biogenic Amine Production by Bacterial Strains from Kimchi and Fermented Seafood Products

Microorganisms play a major role in the production of BA during fermentation through the decarboxylation of free amino acids. LAB responsible for fermentation have been reported to produce putrescine, cadaverine, histamine, and tyramine [15]. Table 3 displays the BA production by bacterial strains isolated from various kimchi and fermented seafood products. Tsai et al. [27] reported that LAB strains isolated from kimchi products purchased from Taiwanese markets were capable of producing histamine and other BA. The isolated strains identified as *Lactobacillus paracasei* subsp. *paracasei*, *Lb*. *brevis*, and *Brevibacillus brevis* were tested for *β*-phenylethylamine, putrescine, cadaverine, histamine, and spermine production in assay media. The reported results showed that *Lb*. *paracasei* subsp. *paracasei*, *Lb*. *brevis*, and *Bb*. *brevis* produced histamine at concentrations of 15.1, 13.6, and 16.3–43.1 µg/mL, respectively. Other BA were detected at concentrations lower than 15 µg/mL. Kim and Kim [53] isolated LAB strains from kimchi identified as *Lb*. *brevis*, *Lb*. *curvatus*, *Leuconostoc mesenteroides*, and *Staphylococcus hominis* that demonstrated tyramine production capabilities at over 200 µg/mL in assay media. Jeong and Lee [54] reported on putrescine, cadaverine, histamine, and tyramine production in assay media by LAB isolated from kimchi including *Leu*. *citreum*, *Leu*. *lactis*, *Leu*. *mesenteroides*, *Weissella cibaria*, *W*. *confusa*, and *W*. *paramesenteroides*. The results revealed that *Leuconostoc* spp. did not produce histamine and tyramine, however, putrescine and cadaverine were produced at concentrations lower than 20 µg/mL. *Weissella* spp. also produced putrescine and cadaverine at concentrations lower than 20 µg/mL, however, some strains produced histamine and tyramine at concentrations higher than 50 µg/mL. Compared to *Leuconostoc* spp., *Weissella* spp. produced a wider variety of BA at higher concentrations, prompting recommendations for stricter safety guidelines for screening starter *Weissella* strains suitable for kimchi fermentation [54].

Other varieties of kimchi were also reported to contain microorganisms capable of BA production. While the majority of the LAB strains isolated from *Chonggak* kimchi and *Kkakdugi* did not produce BA at detectable levels, some isolated LAB strains reportedly produced tyramine in the ranges of 260.93–339.56 µg/mL and 287.23–386.17 µg/mL, respectively, in BA production assay media [30]. Aside from tyramine, other BA were not detected in the same assay media. Although the study did not specify the bacterial species capable of producing BA, *Lb*. *brevis* was suggested as a strong producer of BA. Lee et al. [31] reported the BA production in assay media by LAB strains isolated from *Gat* kimchi and *Pa* kimchi. From *Gat* kimchi, *Enterococcus faecium*, *Lb*. *brevis*, and *Leu*. *mesenteroides* produced the highest concentrations of tyramine in the ranges of 259.10–269.57 µg/mL, ND-365.96 µg/mL, and 145.14–301.67 µg/mL, respectively. *Lb*. *brevis* strains also produced putrescine ranging from ND to 320.42 µg/mL. From *Pa* kimchi, the isolated LAB strains identified as *Lb*. *brevis* and *Lb*. *sakei* produced the highest concentration of BA such as tyramine in the ranges of ND-301.52 µg/mL and 113.98–131.36 µg/mL, respectively. Also, a *Lb*. *brevis* strain produced putrescine at 362.44 µg/mL. Aside from putrescine and tyramine, other BA produced by LAB strains were reported at concentrations lower than 60 µg/mL. Based on the reported BA production capabilities of isolated strains, LAB appear to contribute to the BA content in kimchi, especially tyramine which were produced at the highest concentrations.

Aside from LAB, other bacterial species isolated from *Jeotgal* products were reported to have BA production capabilities. *S. equorum* strains isolated from *Saeu*-*jeot* and *Myeolchi*-*jeot* were reported to be capable of producing putrescine, cadaverine, histamine, and tyramine in assay media [55,56]. The reported results showed that all BA were detected at concentrations below 50 µg/mL. Lim [57] isolated bacterial strains from *Myeolchi*-*jeot* which were identified as *Bacillus licheniformis*, *Serratia marcescens*, *S. xylosus*, *Aeromonas hydrophila*, and *Morganella morganii*, and the strains were capable of producing high concentrations of histamine in assay media at 1699.3 ± 35.6 µg/mL, 1987.2 ± 27.8 µg/mL, 2257 ± 30.7 µg/mL, 1655.5 ± 41.2 µg/mL, and 2869.4 ± 49.0 µg/mL, respectively. Mah et al. [58] suggested that *Bacillus* species, especially *B*. *licheniformis*, contributed towards BA content as the isolated strains isolated from *Myeolchi*-*aekjeot* were capable of producing putrescine, cadaverine, histamine, and tyramine. Thus, the isolated bacterial strains appear to contribute to the high histamine content of fermented seafood products, which in turn contribute to the BA content of kimchi.

The aforementioned studies reported BA production by isolated strains at widely varying concentrations, even among the same species. Lee et al. [31] suggested that the BA production by LAB isolated from kimchi may be strain-dependent. Differences in BA production are widely considered to be strain-dependent, and not species-dependent [59]. The claim is further substantiated by the evidence for horizontal gene transfer for decarboxylase genes [60,61,62]. For example, as tyrosine decarboxylation was observed only for some strains, even belonging to the same species of LAB, tyramine production is considered strain-specific rather than species-specific [63]. Nonetheless, BA production by isolated strains indicates a risk for BA accumulation during *Jeotgal* and kimchi fermentation. Consequently, the control of BA accumulation during the production of fermented foods necessitates the reduction of microbial BA production by control of fermentation conditions, utilization of starter cultures, and sanitary practices to prevent contamination by BA-producing microorganisms.

## 5. Strategies to Reduce Biogenic Amine Content in Kimchi Products

Despite the risks associated with BA accumulation, limited research has been conducted on reducing the BA content of kimchi products. Instead of directly reducing BA content in kimchi, several studies have reported various methods to reduce BA concentrations in the kimchi ingredients *Jeotgal* and *Aekjeot*. Kim et al. [64] reported that kimchi produced using fermented seafood products contained BA at significantly higher concentrations. Lee et al. [65] suggested that the BA concentration of kimchi products may be reduced by limiting the quantity of the fermented seafood products used during kimchi production. For example, Kang [66] reported the histamine content of kimchi without *Myeolchi*-*aekjeot* at safe levels, however, the addition of *Myeolchi*-*aekjeot* raised histamine content to unsafe concentrations above the recommended limit by a factor of approximately 6. The study also described the effect of heat treatment of *Myeolchi*-*aekjeot* on the histamine content of kimchi. Histamine concentrations in kimchi produced using heat-treated *Myeolchi*-*aekjeot* were reported at 546.14 ± 1.33 mg/kg, while non-treated kimchi contained 592.78 ± 3.43 mg/kg. The reported results indicate that microorganisms from *Myeolchi*-*aekjeot* contributed towards the production of histamine during kimchi fermentation. Also, as research shows that histamine is heat-stable [67], the lower BA content in kimchi produced using the heat-treated *Myeolchi*-*aekjeot* may be due to the sterilization of histamine-producing microorganisms [66]. In addition to the contribution of BA content in kimchi by *Myeolchi*-*aekjeot*, Lee et al. [31] suggested that microorganisms from *Myeolchi*-*aekjeot* may produce BA during kimchi fermentation. Utilizing substitute ingredients in lieu of *Myeolchi*-*aekjeot* and *Kkanari*-*aekjeot* may also be effective in reducing BA content in kimchi. As other *Jeotgal* products including *Ojingeo*-*jeot* (salted and fermented sliced squid), *Toha*-*jeot* (salted and fermented *toha* shrimp), *Jogae*-*jeot* (salted and fermented clam), *Baendaengi*-*jeot* (salted and fermented big-eyed herring), and *Eorigul*-*jeot* (salted and fermented oysters) have been found to contain individual BA content below 100 mg/kg [47], utilization of the fermented seafood products with low BA content for kimchi production is expected to reduce the overall BA content of kimchi products [29].

Research on using additives to reduce the BA content of fermented seafood products has also been reported. Mah et al. [68] conducted research to reduce BA production by microorganisms isolated from *Myeolchi*-*jeot*, introducing additives into assay media and *Myeolchi*-*jeot*. The results confirmed that compared to the control, garlic extract was the most effective inhibitor of bacterial growth and BA production by yielding lower in vitro production of putrescine, cadaverine, histamine, tyramine, and spermidine by 11.2%, 18.4%, 11.7%, 30.9%, and 17.4%, respectively. Further results revealed that compared to *Myeolchi*-*jeot* samples treated with ethanol (control), the addition of 5% garlic extract to *Myeolchi*-*jeot* (treatment) inhibited bacterial growth and consequently reduced overall BA production by up to 8.7%. In another study by Mah and Hwang [69], other additives were also used for the reduction of BA production by *Myeolchi*-*jeot* microorganisms in assay media and *Myeolchi*-*jeot*. Among the additives tested in assay media, glycine most effectively inhibited in vitro BA production by bacterial strains. In comparison to the control without additives, the addition of 10% glycine in assay media resulted in reductions in putrescine, cadaverine, histamine, tyramine, and spermidine production by 32.6%, 78.4%, 93.2%, 100.0%, and 100.0%, respectively. Compared to the *Myeolchi*-*jeot* samples salted at 20% NaCl, additional supplementation of 5% glycine reportedly reduced overall BA content by 73.4%. The results suggest that the addition of glycine as well as salt may improve the safety of fermented seafood products. It is noteworthy that despite the results showing effective BA reduction, the use of garlic extract or glycine may affect the flavor of the final product.

Aside from additives, other studies have utilized starter cultures to reduce BA content in *Jeotgal*. In a study by Mah and Hwang [70], some bacterial strains isolated from *Myeolchi*-*jeot* were found to reduce BA content in *Myeolchi*-*jeot*. The reported results showed that, of the 7 starter candidate strains, *S*. *xylosus* exhibited the highest histamine degradation capability as well as the ability to slightly degrade tyramine in assay media. In comparison to the uninoculated *Myeolchi*-*jeot* control, the addition of the starter culture reduced the production of putrescine, cadaverine, histamine, tyramine, and spermidine by 16.5%, 10.8%, 18.0%, 38.9%, and 45.6%, respectively. Jeong et al. [56] isolated strains from *Jeotgal* for use as potential starters and found that *S. equorum* strain KS1039 did not produce putrescine, cadaverine, histamine, and tyramine in vitro.

A limited number of studies have even attempted to directly reduce the BA content of kimchi through the inoculation of bacterial strains. Kim et al. [71] reported reductions in tryptamine, putrescine, cadaverine, histamine, and tyramine levels in *Baechu* kimchi fortified with *Leu. carnosum*, *Leu. mesenteroides*, *Lb. plantarum*, and *Lb. sakei* strains. Similarly, Jin et al. [30] reported that *Kkakdugi* and *Chonggak* kimchi inoculated with *Lb*. *plantarum* strains incapable of producing BA contained lower level of tyramine (but not the other BA) than the uninoculated control. Therefore, utilizing LAB strains unable to produce (and/or able to degrade) BA as kimchi starter cultures may likely reduce the total BA content during kimchi fermentation.

Although the aforementioned studies have shown both direct and indirect methods of reducing BA content in kimchi, current commercial kimchi production processes do not appear to utilize the BA reduction techniques. This might be due to the application of BA reduction methods such as the use of additives, starter cultures, and adjusting the quantity of fermented seafood products have been reported to affect the flavor of kimchi products [69,72,73]. Consequently, inconsistent product quality is reflected in the wide range of BA content of kimchi products, including concentrations that exceed recommended limits for safe consumption. The high BA content reported for various kimchi products indicates that modern production methods require further preventative measures to ensure the safety of the fermented vegetable products, including practical application of research-based BA reduction techniques described above. Commercial kimchi production may greatly benefit from utilizing the aforementioned and novel strategies including control of fermentation conditions, utilizing starter cultures, alternative ingredients, and/or ingredients with low BA content. Furthermore, the establishment and expansion of regulations limiting BA content in fermented foods remain necessary to safeguard consumers against the potential BA intoxication.

## 6. Conclusions

The current study evaluated the BA content of kimchi, a term used to describe a group of Korean fermented vegetable products. Some kimchi samples have been reported to contain high concentrations of BA which exceeded recommended limits. Consumption of the fermented foods with high BA content may have detrimental effects on the body. Several factors contribute to the high BA concentrations in kimchi, which include BA production by microorganisms during fermentation and BA content of ingredients such as *Jeotgal* and *Aekjeot*. As variables such as ingredients, microorganisms, and initial BA content of *Jeotgal* that influence kimchi fermentation differed extensively, the reported BA concentrations of kimchi products also varied widely, even among the same varieties. Due to the large variations among kimchi products, standardization of kimchi production appears to be necessary to limit BA content. Furthermore, though several studies have described methods to indirectly reduce BA concentrations in kimchi by reducing the BA content of ingredients *Jeotgal* and *Aekjeot*, limited research has been conducted on the direct reduction of BA content in kimchi products. To ensure the safe consumption of kimchi products, further research on methods to reduce the BA concentrations below recommended limits appears to be necessary. In conjunction with BA reduction studies, implementation of regulations such as continuous monitoring during production remains necessary to control BA content in kimchi and *Jeotgal* products.

## Figures and Tables

**Table 1:** Biogenic amine content of Korean fermented vegetable products.

Korean Fermented Vegetable Products	N ^1^	Biogenic Amines (mg/kg) ^2^								Ref.
		TRP	PHE	PUT	CAD	HIS	TYR	SPD	SPM	
*Baechu* kimchi (Napa cabbage kimchi)	3	NT ^3^	NT	11.2–89.0 ^4^	ND ^5^-151.8	ND-5.1	ND-28.2	ND	ND	[29]
	20	2.3–22.6	ND-6.8	15.1–240.4	3.6–44.9	0.6–142.3	9.7–118.2	7.7–16.5	ND-3.7	[25]
	37	ND-114	ND	ND-73	ND-1550	ND-5350	ND-42	ND-88	ND-121	[27]
	18	ND-43.9	NT	ND-245.9	ND-63.3	NT	ND-103.6	ND-74.8	NT	[26]
	20	ND-74.8	ND-2.0	2.3–148.6	0.9–39.8	ND-21.8	1.1–27.9	ND-6.7	ND-5.1	[28]
*Baek* kimchi (Napa cabbage kimchi prepared without red pepper powder)	3	NT	NT	ND-54.7	ND-94.8	ND	ND	ND	ND	[29]
	3	tr ^6^	NT	1.9–39.6	11.5–25.6	NT	7.8–64.9	ND-1.7	NT	[26]
*Chonggak* kimchi (ponytail radish kimchi)	3	NT	NT	ND-11.2	ND-70.7	ND	ND	ND	ND	[29]
	3	2.3–15.2	NT	ND-20.3	ND-85.7	NT	20.2–58.1	ND	NT	[26]
	5	ND-23.70	ND-2.80	3.89–853.70	2.00–148.50	8.24–131.20	0.79–18.70	6.10–14.00	ND-20.74	[30]
*Gat* kimchi (mustard leaf kimchi)	3	NT	NT	ND-10.4	ND-11.6	ND	ND	ND	ND	[29]
	13	ND-26.74	ND-15.75	1.89–720.82	2.12–52.43	3.30–232.10	1.28–149.77	12.26–32.62	ND-61.94	[31]
*Godeulppaegi* (Korean lettuce kimchi)	3	NT	NT	ND-6.4	ND-26.7	ND	ND	ND	ND	[29]
*Kkakdugi* (diced radish kimchi)	3	NT	NT	ND-15.4	ND-55.1	ND	ND-9.0	ND	ND	[29]
	5	5.5–18.6	NT	ND-51.6	ND-56.2	NT	ND-10.8	ND-21.8	NT	[26]
	5	ND	ND-15.24	10.85–982.32	ND-124.60	18.75–127.78	2.97–76.95	ND-16.76	ND-3.10	[30]
*Pa* kimchi (green onion kimchi)	3	NT	NT	ND-7.8	ND-15.9	ND-21.7	ND	ND	ND	[29]
	13	ND-15.95	ND-5.97	ND-254.47	ND-123.29	8.67–386.03	ND-181.10	2.32–18.74	ND-33.84	[31]
*Yeolmu* kimchi (young radish kimchi)	3	NT	NT	ND	ND	ND	ND	ND	ND	[29]

^1^ N: Number of samples examined; ^2^ TRP: tryptamine, PHE: *β*-phenylethylamine, PUT: putrescine, CAD: cadaverine, HIS: histamine, TYR: tyramine, SPD: spermidine, SPM: spermine; ^3^ NT: not tested; ^4^ Values are the minimum and maximum concentrations reported. The same number of digits is used after the decimal point in the values, as was presented in the corresponding references; ^5^ ND: not detected; ^6^ tr: trace.

**Table 2:** Biogenic amine content of Korean fermented seafood products.

Korean Fermented Seafood Products	N ^1^	Biogenic Amines (mg/kg) ^2^								Ref.
		TRP	PHE	PUT	CAD	HIS	TYR	SPD	SPM	
*Myeolchi*-*jeot* (salted and fermented anchovy)	3	NT ^3^	NT	92–241 ^4^	ND ^5^-665	155–579	63–244	ND-43	ND-77	[47]
*Myeolchi*-*aekjeot* (salted and fermented anchovy sauce)	4	NT	NT	86.1–178.9	ND	684.6–1154.7	222.6–383.1	ND-358.6	ND	[29]
	8	60.1–296.8	9.3–54.1	33.8–182.1	81.6–263.6	352.5–1127.6	93.9–611.3	4.7–27.1	1.9–12.2	[25]
	15	ND-382.2	ND-85.3	ND-680.0	ND-126.1	684.5–1205.0	77.5–381.1	ND	ND	[48]
	10	NT	NT	NT	NT	584.59–2070.58	NT	NT	NT	[49]
	12	NT	NT	NT	NT	150–1112	NT	NT	NT	[50]
	5	35.0–193.5	20.0–36.9	41.8–173.3	100.0–253.0	196.0–393.2	211.4–446.0	0.8–6.7	1.4–4.1	[28]
*Kkanari*-*aekjeot* (salted and fermented sand lance sauce)	4	NT	NT	55.5–136.4	ND-2.8	308.2–959.7	131.0–203.1	ND-30.9	ND	[29]
	8	62.0–187.2	10.4–51.7	1.6–311.6	52.1–314.8	215.4–1124.1	142.7–583.0	4.0–23.4	2.2–12.8	[25]
	16	ND-410.0	ND-17.9	ND-674.3	ND-96.9	308–732.2	112.3–328.0	ND	ND	[48]
	10	NT	NT	NT	NT	194.01–1839.68	NT	NT	NT	[49]
	5	122.5–242.5	18.3–32.5	30.8–43.8	52.5–168.3	183.4–1038.9	155.7–252.4	3.4–6.4	1.2–5.6	[28]
*Saeu*-*jeot* (salted and fermented shrimp)	2	NT	NT	ND	ND	ND	ND	ND	33–62	[47]
	5	5.3–10.6	ND-1.9	2.0–5.2	6.7–8.5	28.6–33.0	11.2–15.2	1.6–2.9	ND–0.7	[25]
	8	11.8–14.5	5.3–10.6	5.2–12.4	6.7–8.5	28.6–32.0	ND-45.5	ND	ND	[48]
	5	3.3–8.1	ND-4.2	2.8–5.4	ND-1.5	2.3–12.7	1.4–7.4	ND-0.8	0.4–9.6	[28]

^1^ N: Number of samples examined; ^2^ TRP: tryptamine, PHE: *β*-phenylethylamine, PUT: putrescine, CAD: cadaverine, HIS: histamine, TYR: tyramine, SPD: spermidine, SPM: spermine; ^3^ NT: not tested; ^4^ Values are the minimum and maximum concentrations reported. The same number of digits is used after the decimal point in the values, as was presented in the corresponding references; ^5^ ND: not detected.

**Table 3:** Biogenic amine production by bacterial strains isolated from Korean fermented vegetable and seafood products.

Korean Fermented Vegetable and Seafood Products	Strains	N ^1^	Biogenic Amines (µg/mL) ^2^								Ref.
			TRP	PHE	PUT	CAD	HIS	TYR	SPD	SPM	
*Baechu* kimchi (Napa cabbage kimchi)	*Lactobacillus paracasei* subsp. *paracasei*	1	NT ^3^	ND ^4^	ND	0.3	15.1	NT	NT	4.5	[27]
	*Lactobacillus brevis*	1	NT	4.3	0.2	0.8	13.6	NT	NT	5.6	
	*Brevibacillus brevis*	2	NT	ND-3.8 ^5^	ND-0.1	ND-11.2	16.3–43.1	NT	NT	6.8–8.8	
	*Lactobacillus brevis*	6	NT	NT	NT	NT	NT	287–372	NT	NT	[53]
	*Lactobacillus curvatus*	4	NT	NT	NT	NT	NT	333–388	NT	NT	
	*Leuconostoc mesenteroides*	2	NT	NT	NT	NT	NT	282–322	NT	NT	
	*Staphylococcus hominis*	2	NT	NT	NT	NT	NT	287–296	NT	NT	
	*Leuconostoc citreum*	2	NT	NT	ND	18.1–18.2	ND	ND	NT	NT	[54]
	*Leuconostoc lactis*	4	NT	NT	15.6–16.2	ND	ND	ND	NT	NT	
	*Leuconostoc mesenteroides*	3	NT	NT	ND	17.6–19.1	ND	ND	NT	NT	
	*Weissella cibaria*	16	NT	NT	ND-17.7	ND-18.8	ND-72.9	ND-59.9	NT	NT	
	*Weissella confusa*	8	NT	NT	ND-17.1	ND-19.5	ND-73.3	ND-56.6	NT	NT	
	*Weissella paramesenteroides*	1	NT	NT	ND	ND	55.2	56.3	NT	NT	
*Kkakdugi* (diced radish kimchi)	Lactic acid bacteria	39	ND	ND	ND	ND	ND	287.23–386.17	ND	ND	[30]
*Chonggak* kimchi (ponytail radish kimchi)	Lactic acid bacteria	16	ND	ND	ND	ND	ND	260.93–339.56	ND	ND	
*Pa* kimchi (green onion kimchi)	*Lactobacillus brevis*	14	ND	ND-2.39	ND-362.44	ND-54.79	ND	ND-301.52	ND	ND	[31]
	*Lactobacillus sakei*	2	ND	1.00–3.96	ND	ND	ND	113.98–131.36	ND	ND	
*Gat* kimchi (mustard leaf kimchi)	*Enterococcus faecium*	2	ND	3.51–3.88	ND	ND	ND	259.10–269.57	ND	ND	
	*Lactobacillus brevis*	7	ND	ND-2.34	ND-320.42	ND-47.73	ND	ND-365.96	ND	ND	
	*Leuconostoc mesenteroides*	2	ND	1.47–1.91	ND	ND	ND	145.14–301.67	ND	ND	
*Myeolchi*-*jeot* (salted and fermented anchovy) *Saeu*-*jeot* (salted and fermented shrimp)	*Staphylococcus equorum*	39	NT	NT	ND-22.6	ND-29.6	ND-40.0	ND-29.7	NT	NT	[56]
*Myeolchi*-*jeot* (salted and fermented anchovy)	*Bacillus licheniformisr*	1	NT	NT	NT	NT	1699.3 ± 35.6 ^6^	NT	NT	NT	[57]
	*Serratia marcescens*	1	NT	NT	NT	NT	1987.2 ± 27.8	NT	NT	NT	
	*Staphylococcus xylosus*	1	NT	NT	NT	NT	2257.4 ± 30.7	NT	NT	NT	
	*Aeromonas hydrophila*	1	NT	NT	NT	NT	1655.5 ± 41.2	NT	NT	NT	
	*Morganella morganii*	1	NT	NT	NT	NT	2869.4 ± 49.0	NT	NT	NT	

^1^ N: Number of samples examined; ^2^ TRP: tryptamine, PHE: *β*-phenylethylamine, PUT: putrescine, CAD: cadaverine, HIS: histamine, TYR: tyramine, SPD: spermidine, SPM: spermine; ^3^ NT: not tested; ^4^ ND: not detected; ^5^ Values are the minimum and maximum concentrations reported. The same number of digits is used after the decimal point in the values, as was presented in the corresponding references; ^6^ mean ± standard deviation. The same number of digits is used after the decimal point in the values, as was presented in the corresponding references.

## Supplementary Materials

The following are available online at https://www.mdpi.com/2304-8158/8/11/547/s1, Table S1: Biogenic amine content of fermented vegetable and seafood products from various countries.
