# Supplementary material for: Occurrence and Reduction of Biogenic Amines in Kimchi and Korean Fermented Seafood Products
Source: Foods. 2019 Nov 4;8(11):547. doi: 10.3390/foods8110547 (PMC6915361; doi:10.3390/foods8110547)
Supplement: Supplementary file 1 [file foods-08-00547-s001.pdf]

Table S1. Biogenic amine content of fermented vegetable and seafood products from various countries.

Vegetable and Fermented Seafood Products	N [1]	Biogenic Amines (mg/kg) [2]								Ref.
		TRP	PHE	PUT	CAD	HIS	TYR	SPD	SPM	
Sauerkraut (fermented white cabbage)	50	NT [3]	NT	NT	NT	9.1–130 [4]	NT	NT	NT	[35]
	8	NT	0–9	6–550	1–311	1–104	2–192	NT	NT	[15]
Tsukemono (fermented, salted, and/or pickled vegetables)	39	NT	NT	0.4–61.7	ND [5]-28.9	ND-264	ND-369	ND-17.2	NT	[37]
Mustard pickle (salted and fermented mustard green)	37	ND-155	ND	ND-131	ND-24	ND-89	ND-43	ND	ND-95	[38]
Cincalok (salted and fermented shrimp)	10	ND-25.3	NT	10.6–803.4	NT	6.8–257.8	156.1–681.2	ND-2.2	NT	[51]
Budu (salted and fermented anchovy)	8	ND-317.6	NT	28.4–64.9	NT	99.0–372.9	58.0–852.6	3.0–7.0	NT	[51]
	12	NT	NT	NT	NT	222.1–1064.0	NT	NT	NT	[52]

[1] N: Number of samples examined; [2] TRP: tryptamine, PHE: *β*-phenylethylamine, PUT: putrescine, CAD: cadaverine, HIS: histamine, TYR: tyramine, SPD: spermidine, SPM: spermine; [3] NT: not tested; [4] Values are the minimum and maximum concentrations reported. The same number of digits is used after the decimal point in the values, as was presented in the corresponding references; [5] ND: not detected.
